# Complete Genome Sequence of Middle East Respiratory Syndrome Coronavirus (MERS-CoV) from the First Imported MERS-CoV Case in China

**DOI:** 10.1128/genomeA.00818-15

**Published:** 2015-08-13

**Authors:** Roujian Lu, Yanqun Wang, Wenling Wang, Kai Nie, Yanjie Zhao, Juan Su, Yao Deng, Weimin Zhou, Yang Li, Huijuan Wang, Wen Wang, Changwen Ke, Xuejun Ma, Guizhen Wu, Wenjie Tan

**Affiliations:** a Key Laboratory of Medical Virology, Ministry of Health, National Institute for Viral Disease Control and Prevention, China CDC, Beijing, China; b Center for Disease Control and Prevention of Guangdong Province, Guangzhou, China

## Abstract

On 26 May 2015, an imported Middle East respiratory syndrome coronavirus (MERS-CoV) was identified in Guangdong Province, China, and found to be closely related to the MERS-CoV strain prevalent in South Korea. The full genome of the ChinaGD01 strain was sequenced and analyzed to investigate the epidemiology and evolution of MERS-CoV circulating in South Korea and China.

## GENOME ANNOUNCEMENT

Middle East respiratory syndrome coronavirus (MERS-CoV) is a new member of beta coronavirus lineage C. Different from severe acute respiratory syndrome (SARS) coronavirus and the common-cold coronavirus, most confirmed cases of MERS-CoV have displayed the symptoms of severe acute respiratory illness, with a mortality rate of 37% (1, 2). Since the first confirmed case in Saudi Arabia in 2012, the epidemic situation of MERS-CoV showed sporadic outbreaks in local areas, especially in the Arabian Peninsula (3). The source of MERS-CoV is currently unknown, although it is likely to have originated from *Camelus* dromedaries or bats (4–6). In 20 May 2015, the first MERS-CoV case was found in South Koreans who had traveled to Middle Eastern countries. As of 15 June 2015, the MERS-CoV was spreading in South Korea, with 126 laboratory-confirmed cases (including 10 deaths). In addition, one of the cases was a South Korean national who traveled to Guangdong Province and was diagnosed as the first imported MERS-CoV case in China (7). To determine the genetic relationship of the first imported strain ChinaGD01 and other global MERS-CoV strains, the complete genome of the ChinaGD01 strain was sequenced and analyzed.

In this study, viral RNA was extracted from the nasopharyngeal swabs using the QIAamp viral RNA minikit. Overlapping reverse transcription-PCR special primers were conducted by 44 sets of special primers designed according to all available published complete genomes of MERS-CoV. The 5′ and 3′ ends of the genome of ChinaGD01 were determined by rapid amplification of cDNA ends. Meanwhile, the original respiratory sample was also processed for deep sequencing using the Ion Torrent PGM sequencer system.

The first imported MERS-CoV strain was named ChinaGD01 and was 30,114 nucleotide (nt) long, including the 3′- and 5′-UTRs. The ChinaGD01 strain shows the typical betacoronavirus organization: a 5′ untranslated region (UTR) (nt 1 to 272), replicase complex (open reading frame 1ab [ORF1ab], nt 273 to 21508), S gene (nt 21450 to 25511), ORF3 (nt 25526 to 25837), ORF4a (nt 25846 to 26175), ORF4b (nt 26087 to 26827), ORF5 (nt 26834 to 27508), E gene (nt 27584 to 27832), M gene (nt 27847 to 28506), N gene (nt 28560 to 29801), ORF8b gene (nt 28756 to 29094), and 3′ UTR (nt 29094 to 30114). Alignment of the full genome of strain ChinaGD01 and other available genomes of MERS-CoV showed the closest genetic relatedness with the first South Korean genome (GenBank accession number KT029139.1) of MERS-CoV (99.96% nt identify), followed by the latest strain (KT026453), prevalent in Saudi Arabia (99.92% nt identify). The genome of ChinaGD01 has 2 amino acid differences in the S gene and 3 amino acid differences from the South Korean strain (KT029139.1) in the ORF1ab gene. There were no nucleotide insertions or deletions between ChinaGD01 and other MERS-CoV strains.

This is the first complete genome of an isolate from an imported MERS-CoV case in China. Our sequence data will provide further insight into the evolution and genetic diversity of MERS-CoV prevalent in South Korea and China.

### Nucleotide sequence accession number.

The complete genome of the ChinaGD01 strain was deposited at GenBank under the accession number KT006149.
